# Identification of Leaf Rust Resistance Genes in Selected Wheat Cultivars and Development of Multiplex PCR

**DOI:** 10.1515/biol-2019-0036

**Published:** 2019-07-22

**Authors:** Agnieszka Tomkowiak, Roksana Skowrońska, Alicja Buda, Danuta Kurasiak-Popowska, Jerzy Nawracała, Przemysław Łukasz Kowalczewski, Mateusz Pluta, Dominika Radzikowska

**Affiliations:** 1 Department of Genetic and Plant Breeding, Faculty of Agronomy and Bioengineering, Poznań University of Life Sciences, 11 Dojazd Str, 60-632 Poznań, Poland; 2 Institute of Food Technology of Plant Origin, Faculty of Food Science and Nutrition, Poznań University of Life Sciences, 31 Wojska Polskiego Str, 60-624 Poznań, Poland; 3 Department of Agronomy, Faculty of Agronomy and Bioengineering, Poznań University of Life Sciences, 11 Dojazd Str, 60-632 Poznań, Poland

**Keywords:** Leaf rust, multiplex PCR, SSR molecular markers, wheat

## Abstract

Ten leading wheat cultivars originating from the Plant Breeding and Acclimatization Institute (IHAR) - National Research Institute (Poland) and the Department of Gene Bank (Czech Republic) were used to establish a field experiment in 2017 and 2018 at the Dłoń Experimental Farm. The analyzed wheat genotypes were characterized by diversified field resistance to leaf rust. Jubilatka, Thatcher and Sparta were the most resistant cultivars in field conditions in both 2017 and 2018. The aim of the work was to identify the *Lr11, L13, Lr16* and *Lr26* genes encoding resistance to leaf rust using molecular SSR markers (*wmc24, wmc261, Xgwm630, Xwmc764* and *P6M12*) and to develop multiplex PCR conditions to accelerate identification of these genes. Markers of three leaf rust resistance genes have been identified simultaneously in these cultivars. Jubilatka, Thatcher and Sparta cultivars may serve as a good source of the analyzed leaf rust resistance genes. In addition, multiplex PCR conditions have been developed for the simultaneous identification of the *Lr11* and *Lr16* and *Lr11* and *Lr26* gene pairs.

## 1 Introduction

Common wheat (*Triticum aestivum ssp. vulagare*) is the most commonly cultivated plant based on the surface area of crops. Wheat is mainly grown for consumption and industrial purposes [1, 2]. In 2017, cereals in the European Union covered 56 million ha from which nearly 300 million tons of grain were harvested. In Poland, the area of wheat cultivation amounted to almost 2.5 million ha while the average yield was almost 5 tons from one ha [3].

Leaf rust caused by *Puccinia recondita f. sp. tritici* and stripe (yellow) rust caused by *Puccinia striiformis f. sp. tritici* are the most common types of rust that affect cereal crops in Poland. Losses in the yield that resulting from grain heterogeneity caused by this disease, amount to 15%, but may reach even 30-60% under favorable conditions [4]. Wheat genes responsible for resistance to pathogens and pests are divided into 25 classes and described in the Catalogue of Gene Symbols for Wheat. The largest group constitutes of genes that determine resistance to leaf rust. Currently, 88 *Lr* (leaf rust) genes that encode the resistance to leaf rust have been identified [5]. Most of them are R genes (Major Resistance Genes) that determine monogenic resistance according to the Flor ‘gene-for-gene’ theory [6] in which pathogen avirulence gene products interact directly or indirectly with the main products [7, 8]. In hexaploid wheat, leaf rust resistance genes are widely distributed across the genome, being present on nearly every one of the 42 chromosome arms. Different resistance genes condition characteristic resistance phenotypes or infection types. Race-specific resistance responses such as those conditioned by wheat lines with *Lr3ka*, *Lr3bg* and *Lr11* are manifested by small uredinia surrounded by chlorosis, and lines with *Lr16* have small uredinia surrounded by necrosis. For a number of resistance genes, the expression (dominant or recessive) and number of genes that conditioned avirulence are diferent between different segregating populations of *Puccinia recondita f. sp. tritici*. It can be conditioned by one gene or two genes in different populations at loci which condition avirulence / virulence for *Lr1, Lr3, Lr10, Lr11, Lr29* and *Lr30*. Avirulence in *Puccinia recondita f. sp. Tritici* for *Lr16* and *Lr26* are conditioned by the two dominant genes, and the avirulence for the *Lr10*, *Lr18* and *Lr23* genes is conditioned by a single dominant gene in some segregating populations or recessive genes in other populations [9].

The systematic appearance of new pathogen genotypes is a problem in resistance breeding. *Puccinia recondita f. sp. tritici* is characterized by high genetic variability; it also demonstrates adaptability to climatic conditions, and its spores can migrate over long distances with air currents. For this reason, it is necessary to constantly search for and select sources of resistance to create new resistant wheat cultivars [4, 10]. In plant pathogenic fungi, the mechanism of their variability are: recombination occurring during combining of gametes and meiosis. Variability of pathogens may refer to appearance, structure or function, however, the most important is the variability of pathogenicity. There may be variants more or less pathogenic, variants capable of attacking plants that have not been hosts of a given pathogenic species yet and finally variants capable of attacking specific cultivars of the host plant (so-called variants with specific virulence). According to the theory of complementarity of genes, pathogens have specific virulence genes corresponding to the genes of resistance in plants and are thus able to overcome plant resistance to disease. In recent years a reverse theory has been proclaimed that certain pathogenic variants have avirulence genes, the expression of which is based on inducing immune reactions in plants. The effect of pathogen variability is the instability of plant resistance to disease. The resistance obtained in the bred varieties is overcome by the pathogen due to the emergence of new variants and their dissemination in the population as a result of selective pressure.

Multiplex PCR is one of the techniques that employs molecular markers. The advantage of the method is the possibility of simultaneous application of several primer pairs in the reaction mixture. Because of it, it is possible to identify several markers in one analysis. Markers with a similar primer annealing temperature to the DNA template should be used for their amplification to be possible. This technique allows reduction of the costs of analyses, labor time and the risk of contamination [8]. Tomkowiak et. al. [11] identified the *Pm2, Pm3a, Pm4b* and *Pm6* genes for ten wheat varieties and developed multiplex PCR reaction conditions to reduce the time and limit analysis costs. The following molecular markers were used for gene identification: *Xcfd81, Whs350* and *Xgwm205* (for *Pm2*), *Pm3a* (for *Pm3a*), *STS_241* and *Xgwm382* (for *Pm4b*), *NAU/BCDSTS 135-2* (for *Pm6*).

The aim of the study was to identify the *Lr11*, *Lr13*, *Lr16* and *Lr26* leaf rust resistance genes in 10 wheat cultivars originating from the Plant Breeding and Acclimatization Institute (IHAR) - National Research Institute (Poland) and the Department of Gene Bank (Czech Republic) as well as to develop multiplex PCR conditions to accelerate the identification of these genes.

## 2 Materials and methods

Ten wheat cultivars from Plant Breeding and Acclimatization Institute (IHAR) - National Research Institute (Poland) and the Department of Gene Bank (Czech Republic) that contained leaf rust resistance genes (*Lr11*, *Lr13*, *Lr16* and *Lr26*) were tested (Tab. 1). Field experiment was performed at the Department of Genetic and Plant Breeding Experimental Station housed in the Dłoń Agricultural Experimental Farm of the Poznań University of Life Sciences (51°41’23.835”N 17°4’1.414”E) in 2017 and 2018. Genotypes were sown on 10 m^2^ plots in a randomized block system in triplicate. The assessment of the degree of leaf infection by *Puccinia recondita f. sp. tritici* was carried out in the milky maturity phase (BBCH 71-77) on 40 flag and subflag leaves of randomly selected plants from each plot. The field assessment was carried out in accordance with the recommendations of the European and Mediterranean Plant Protection Organization (EPPO) on the 9° scale (1°-0.1% of infected area, 9°-60% of infected area).

**Table 1:** The degree of resistance to *Puccinia recondita* f. sp. *tritici* infection under field conditions (Dłoń Agricultural Experimental Farm) and the identification of the *Lr11*, *Lr13*, *Lr16* and *Lr26* genes by means of SSR markers.

No.	Genotype	Field conditions		Molecular analysis				
		*Lr11 Wmc24*		*Lr11 Wmc261*	*Lr13 Xgwm630*	*Lr16 Xwmc764*	*Lr26 P6M12*	
		2017	2018					
1	Hussar	5	4	+	+	-	-	+
2	Wilga	8	7	-	+	-	-	-
3	Rialto	6	5	-	+	-	+	+
4	Apollo	7	6	-	+	-	+	-
5	Opata	8	6	+	+	+	-	-
6	Jubilatka	4	2	+	+	-	+	+
7	Thatcher	3	1	-	+	+	+	-
8	Sparta	3	1	-	+	-	+	+
9	Frontana	5	5	-	+	-	-	-
10	Brigadier	7	3	+	+	-	-	+

9° scale: 1°-0.1% of infected area, 9°-60% of infected area “+” indicates the presence of a given DNA fragment characteristic of the marker locus “–” indicates the absence of a given DNA fragment characteristic of the marker locus

DNA was isolated from the leaves of 10-day-old seedlings with the use of Genomic Mini AX PLANT DNA collection kit (A&A Biotechnology, Poland) following the protocol provided with the kit. DNA concentration was determined using a DeNovix spectrophotometer. The samples were diluted with Tris buffer to obtain a uniform concentration of 70 ng μL.

PCR was carried out in a TProfesional Basic gradient thermocycler (Polygen sp. z o.o., Poland). The identification of the *Lr11* gene was carried out with the use of *wmc24* and *wmc261* primers. The following markers were used to determine the presence of the *Lr13*, *Lr16* and *Lr26* genes: *Xgwm630, Xwmc764* and *P6M12*. The primers were synthesized by IDT – Integrated DNA Technologies. The primer sequences were derived from the Grain Genes database (https://wheat.pw.usda.gov) and are presented in Table 2. The 12.75 μL reaction mixture consisted of: water – 5 μL, DreamTaq™ Green PCR Master Mix – 6.26 μL, primers – 2 × 0.25 μL (final concentration was 20 μM), DNA template – 1 μL. After optimization, PCR was carried out under the same conditions regardless of the marker being identified. The profile differed only in the primer annealing temperature, determined based on their melting temperature: initial denaturation for 5 minutes at 94°C, 40 cycles (denaturation – 45 sec at 94°C, primer annealing – 1 min at 53°C, 54°C), 56°C, 57°C, 58°C, synthesis – 1 min at 72°C), final synthesis – 5 min at 72°C, storage at -4°C max. for 24 hours.

**Table 2:** Primer sequences and their annealing temperature in the detection of the *Lr11*, *Lr13*, *Lr16*, *Lr26* genes.

No.	Gene – Marker	Primer sequence	Annealing temperature
1	*Lr11 – wmc24*	F:5’GTGAGCAATTTTGATTATACTG3’	53°C
		R:5’TACCCTGATGCTGTAATATGTG3’	
2	*Lr11 – wmc261*	F:5’GATGTGCATGTGAATCTCAAAAGTA3’	54°C
		R:5’AAAGAGGGTCACAGAATAACCTAAA3’	
3	*Lr13 – Xgwm630*	F:5’GTGCCTGTGCCATCGTC3’	58°C
		R:5’CGAAAGTAACAGCGCAGTGA3’	
4	*Lr16 – Xwmc764*	F:5’ CCTCGAACCTGAAGCTCTGA3’	57°C
		R:5’TTCGCAAGGACTCCGTAACA3’	
5	*Lr26 – P6M12*	F:5’GTACTAGTATCCAGAGGTCACAAG3’	56°C
		R:5’CAGACAAACAGAGTACGGGC3’	

Simultaneous identification of the *Lr11* gene marker (*wmc261*) and the *Lr16* gene marker (*Xwmc764*) was carried out in a volume of 25 μL. Composition of the reaction mixture was as follows: water – 10 μL, DreamTaq MM Master Mix – 12.5 μL, *wmc261* primer – 2 × 0.25 μL, *P6M12* primer – 2 × 0.25 μL, DNA template – 1.5 μL. The reaction was based on the thermal profile specific for the identification of the *Lr16* gene –primer annealing temperature was 57°C.

Simultaneous identification of the *Lr11* (*wmc261*) and *Lr26* gene marker (*P6M12*) was carried out in a volume of 24.9 μL and its composition was as follows: water – 10 μL, DreamTaq MM Master Mix – 12.5 μL, *wmc261* primer – 2 × 0.1 μL, *P6M12* primer – 2 × 0.35 μL, DNA template – 1.5 μL. The reaction was based on the thermal profile specific for the identification of the *Lr26* gene –primer annealing temperature was 56°C.

To visualize the results, PCR products were separated in a 2.5% agarose gel with 1 μL ethidium bromide immersed in TBE 1x for 1 h at 120 V. To visualize PCR products, a Molecular Imager Gel Doc™ XR UV transilluminator was used with the ImageLab™ Software (Bio-Rad, USA).

**Ethical approval**: The conducted research is not related to either human or animals use.

## 3 Results

### 3.1 Field evaluation

Jubilatka, Thatcher and Sparta were the most resistant cultivars in field conditions in both 2017 and 2018. In 2018, infection degree of these cultivars by *Puccinia recondita f. sp. tritici* was only 2° (Jubilatka) and 1° (Thatcher and Sparta), meaning that the infection involved no more than 1% of the leaf area in Jubilatka and 0.1% in Thatcher and Sparta (Tab. 1). These cultivars showed lower resistance in 2017, which was caused by very intense precipitation during the entire growing season, which promoted pathogen development. The least resistant in field conditions were the cultivars Wilga (8° – 2017 and 7° – 2018) and Opata (8° – 2017 and 6° – 2018), in which as many as 45% of the leaf area was infested by *Puccinia recondita f. sp. tritici* in 2017. The remaining cultivars: Hussar, Rialto, Apollo, Frontana, Brigadier were characterized by average resistance to leaf rust both in 2017 and in 2018 (Tab. 1).

### 3.2 Lr11 gene identification

**Figure 1:**
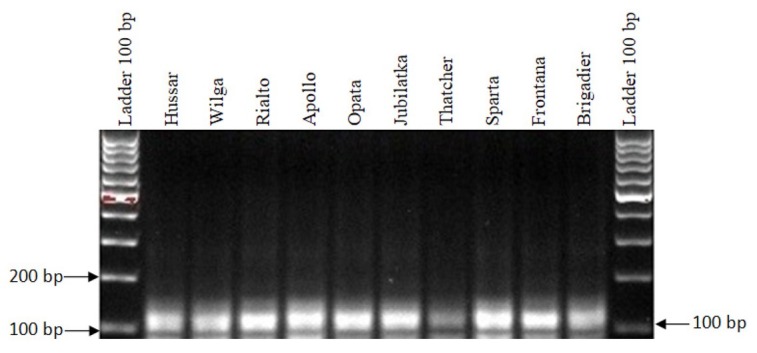
Electropherogram showing the presence of the *wmc261* marker of the *Lr11* gene in wheat cultivars.

A specific product of 152 bp was obtained in the following cultivars as a result of the PCR-SSR analyses carried out using the *wmc24* marker for the *Lr11* gene: Hussar, Opata, Jubilatka and Brigadier. A nonspecific product of 145 bp was obtained in the cultivars Wilga, Rialto, Apollo, Thatcher, Sparta and Frontana, indicating the absence of the *Lr11* gene (Tab. 1). The second tested marker for the *Lr11* gene was *wmc261* that gave a product of 110 bp. The marker was detected in all the analyzed cultivars (Fig. 1, Tab. 1).

### 3.3 Lr13 gene identification

Analysis of the electrophoretic images after the PCR-SSR reaction in which the *Xgwm630* marker linked to the *Lr13* gene was used revealed a specific product of 120 bp in the cultivars Opata and Thatcher (Tab. 1).

### 3.4 Lr16 gene identification

**Figure 2:**
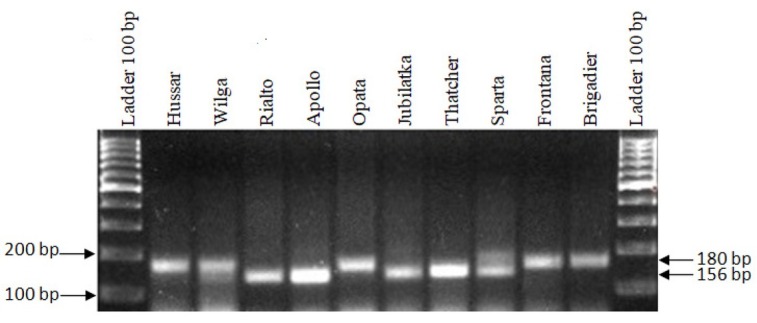
Electropherogram showing the presence of the *Xgwm764* marker of the *Lr16* gene in wheat cultivars.

The *Xwmc764* marker was used to identify the *Lr16* gene. As a result of the analyses, a specific product of 156 bp was amplified in the following cultivars: Rialto, Apollo, Jubilatka, Thatcher and Sparta. A 180-bp product, indicating the absence of the *Lr16* gene, was found in the following cultivars: Hussar, Wilga, Opata, Frontana and Brigadier (Fig. 2., Tab. 1).

### 3.5 Lr26 gene identification

**Figure 3:**
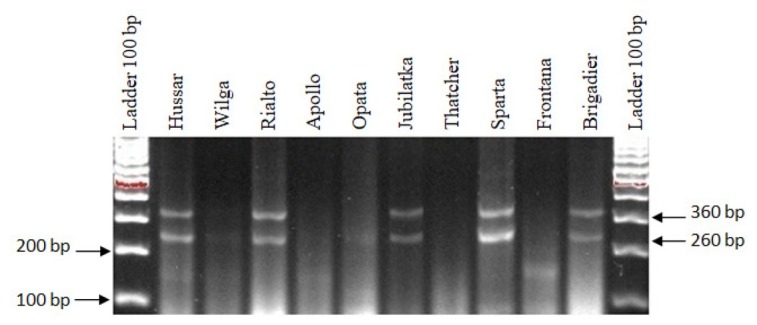
Electropherogram showing the presence of the *P6M12* marker of the *Lr26* gene in wheat cultivars.

The analyses that involved the *P6M12* marker, specific to the *Lr26* gene, resulted in the detection of 260-bp and 360-bp PCR products that indicate the presence of the *Lr26* gene in the following cultivars: Hussar, Rialto, Jubilatka, Sparta and Brigadier. No product was obtained in the cultivars Wilga, Apollo, Opata, Thatcher and Frontana (Fig. 3., Tab. 1).

### 3.6 DNA amplification for the Lr11 and Lr16 genes using the Multiplex PCR method

Two primer pairs, *wmc261* and *Xwmc764*, were used in the PCR reaction. An amplification product of 110 bp was observed in all tested cultivars which proved the presence of the *Lr11* gene. The second marker of the *Lr16* gene confirmed the presence of leaf rust resistance genes in the cultivars Rialto, Apollo, Jubilatka, Thatcher and Sparta in which a product with a size of 156 bp was identified. A non-specific product of 180 bp was obtained in the cultivars Hussar, Wilga, Opata, Frontana and Brigadier.

**Figure 4:**
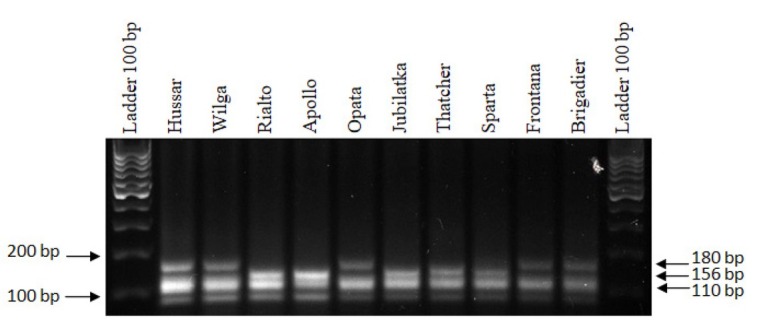
Electropherogram showing the presence of the following markers: *wmc261* of the *Lr11* gene and *Xgwm764* of the *Lr16* gene in wheat cultivars.

The obtained electrophoretic image was reproducible (Fig. 4.)

### 3.7 DNA amplification for the Lr11 and Lr26 genes using the Multiplex PCR method

**Figure 5:**
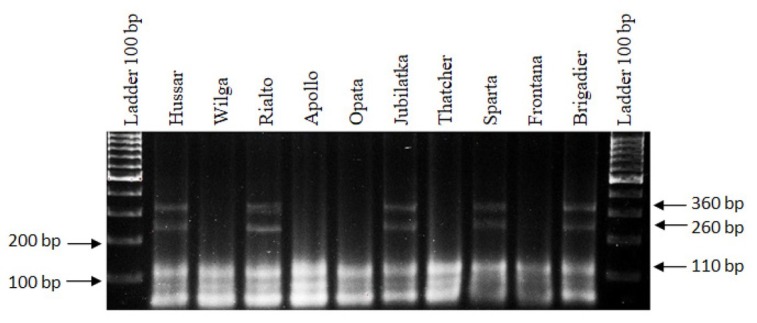
Electrophoregram showing the presence of the following markers: *wmc261* of the *Lr11* gene and *P6M12* of the *Lr16* gene in wheat cultivars.

Three reaction products (110 bp, 260 bp and 360 bp) were obtained after including two primer pairs for the *wmc261* and *P6M12* markers in the PCR reaction in the Hussar, Jubilatka, Sparta and Brigadier cultivars. This indicated the presence of the *Lr11* and *Lr26* genes in these cultivars. One product of 110 bp, specific for the *Lr11* gene marker was detected in the remaining cultivars. The results of the analyses were repeatable (Fig. 5).

## 4 Discussion

Cultivars of winter wheat grown in Poland and newly registered ones are evaluated in terms of resistance to infection by the pathogen *Puccinia recondita f. sp. tritici* [7]. However, after several years, the resistance is often lost. It was found in the USA that 4 years after the introduction of the cultivar ‘Chinese spring’ containing the *Lr9* gene, the resistance was overcome by new pathogen races [12]. Currently, research is conducted on changes in the frequency of virulence genes corresponding to resistance genes [10]. Woźniak-Strzembicka [13] showed that the *Lr9, Lr19, Lr23, Lr24* and *Lr25* genes were effective against the population of the pathogen present in Poland at that time. Liatukas and Ruzgas [14] investigated the effectiveness of the most popular genes in winter wheat occurring in Europe at different growth stages. They showed that the *Lr37* gene was the most effective in the tillering and the adult plant stages and when combined with other genes, while the *Lr13* gene showed efficacy against 29% of the pathogen isolates in the adult stage of the plant. Czajowski et al. [15] conducted a study in 2008-2011 and showed that the *Lr19* gene from *Agropyron elongatum* was still effective against the pathogen causing leaf rust.

The source of the *Lr11* gene is *Triticum aestivum*, originally located on chromosome 2A. Recent research showed that it is located on chromosome 2D [16]. In the present work, linked *wmc24* and *wmc261* markers were used to identify *Lr11*. In the experiments, the *wmc24* marker occurred in the cultivars Hussar, Opata, Jubilatka and Brigadier. The *wmc261* marker was identified in all the analyzed cultivars.

The *Lr13* gene was transferred from *Triticum aestivum* and is located on the short arm of chromosome 2B. It is considered one of the most common genes in wheat cultivars. It is responsible for resistance in the adult plant stage, and its effectiveness is low. The effectiveness of *Lr13* may increase when combined with the *Lr34* or *Lr16* genes. In Australia, *Lr13* and *Lr1* combination provided effective protection against leaf rust. Bansal et al. [17] used the *Lr13* gene and the *Xjsm58* and *Xstm773b* markers to determine the exact location of the *Lr48* gene and proved that these genes were associated and could be used as effective resistance genes in the future. In this study, the *Lr13* leaf resistance gene was identified using the *Xgwm630* marker in the cultivars Opata and Thatcher.

The *Lr16* gene, transferred from *Triticum aestivum*, is widely used and provides partial resistance at the seedling stage. It was also proven to be more effective than other genes at higher temperatures. The best efficiency of this gene was recorded when it was accompanied by the presence of other genes: *Lr13, Lr23* and *Lr34*. Originally, *Lr16* has been assigned to chromosome 4A [18]. Lan et al. [19] investigated the exact location of the *Lr16* gene and proved that it is localized on chromosome 2B. The *YrF* gene, responsible for the resistance to stripe rust (*Puccinia striiformis*), is also located on the arm of this chromosome. For this purpose, the authors used the *Xwmc764* and *Xwmc661* markers. According to the study by Liu et al. [20], the *Xgwm210*, *Xwmc661* and *Xwmc764* markers can be used to identify the *Lr16* gene in wheat breeding programs or to create a pyramid with other genes responsible for leaf rust resistance. In the present work, the *Xwmc764* marker was used to identify the *Lr16* gene, and depending on the cultivar, it was located at a distance of 1.3 and 9 cM from the gene [21]. Moreover, the *Xwmc764* marker of the *Lr16* gene was detected in the following cultivars: Rialto, Apollo, Jubilatka, Thatcher and Sparta.

The *Lr26* gene is derived from rye (*Secale cereale*) and is located on chromosome 1BL/1RS. The translocation of this gene into wheat also resulted in the transfer of resistance genes against stem rust (*Sr31*), stripe rust (*Yr9*) and powdery mildew (*Pm8*) [22]. Hanzalová et al. [23] examined 27 cultivars of winter wheat registered in the Czech Republic for the presence of the *Lr26* and *Lr37* genes. The *Lr26* gene was present only in 4 genotypes, but was linked to the other genes tested. In the current study, the *P6M12* marker was present in the cultivars Hussar, Rialto Jubilatka, Sparta and Brigadier.

The arguments that supports the choice of the multiplex PCR method to detect the presence of multiple genes are low cost of analysis and ease of implementation. De Froidmont [24] used a multiplex PCR method to identify the 1BL/1RS translocation in wheat breeding lines derived from rye lines and to distinguish homozygotes from heterozygotes. Fraaije et al. [25] identified resistance to four pathogens: *Septoria tritici, Stagonospora nodorum*, *Puccinia striiformis* and *Puccinia recondita* using multiplex PCR. They proved that the above method was effective in recognizing genes responsible for the resistance to pathogens at various stages of plant development. In the present work, multiplex PCR was used to simultaneously identify the *Lr11* and *Lr16* and *Lr11* and *Lr26* genes. The results indicate that a multiplex PCR method can be used in breeding programs. Such a method would allow to save time and labor and reduce the cost of analysis.

## 5 Conclusions

Jubilatka, Thatcher and Sparta were the most resistant cultivars in field conditions in both 2017 and 2018. Markers of three leaf rust resistance genes were detected simultaneously in these cultivars. The *Lr11*, *Lr16* and *Lr26* genes were present in Jubilatka and Sparta cultivars, whereas *Lr11*, *Lr13* and *Lr16* in the cultivar Thatcher. Markers of three leaf rust resistance genes were also found in the cultivar Rialto. Nonetheless, this cultivar proved not to be resistant to the pathogen *Puccinia recondita f. sp. tritici* under field conditions. One and two markers of leaf rust resistance genes were detected in Wilga and Opata cultivars, respectively. These cultivars were the least resistant in field conditions. The remaining cultivars: Hussar, Rialto, Apollo, Frontana and Brigadier were characterized by moderate resistance to leaf rust both in 2017 and in 2018. One or two markers of the analyzed *Lr* genes were present in these cultivars. Based on the results, the *c*ultivars Jubilatka, Thatcher and Sparta can be considered a good source of the analyzed leaf rust resistance genes. In addition, the developed multiplex PCR conditions for the simultaneous identification of *Lr11* and *Lr16* as well as *Lr11* and *Lr26* gene pairs can be used in breeding programs in order to shorten the time of molecular analysis.
